# Cutaneous and mucocutaneous leishmaniasis: experience of a Mediterranean hospital

**DOI:** 10.1186/s13071-020-3901-1

**Published:** 2020-01-13

**Authors:** Marta Garrido-Jareño, Antonio Sahuquillo-Torralba, Rabab Chouman-Arcas, Iván Castro-Hernández, José Miguel Molina-Moreno, Margarita Llavador-Ros, María Dolores Gómez-Ruiz, José Luis López-Hontangas, Rafael Botella-Estrada, Miguel Salavert-Lleti, Javier Pemán-García

**Affiliations:** 1 Microbiology Department, La Fe University Hospital, Valencia, Spain; 2 Dermatology Department, La Fe University Hospital, Valencia, Spain; 3 Infectious Diseases Department, La Fe University Hospital, Valencia, Spain; 4 Pathology Department, La Fe University Hospital, Valencia, Spain

**Keywords:** Cutaneous leishmaniasis, Mucocutaneous leishmaniasis, Immune status, microbiological diagnosis, Nucleic acid amplification techniques

## Abstract

**Background:**

Leishmaniasis, considered by the World Health Organization as one of the most important tropical diseases, is endemic in the Mediterranean Basin. The aim of this study was to evaluate epidemiological and clinical characteristics of cutaneous (CL) and mucocutaneous leishmaniasis (MCL) in La Fe University Hospital, Valencia, Spain. The particular focus was on diagnosis techniques and clinical differences according to the immunological status of the patients.

**Methods:**

An eleven-year retrospective observational study of CL and MCL episodes at the hospital was performed. Epidemiological, clinical and therapeutic variables of each case, together with the microbiological and anatomopathological diagnosis, were analyzed.

**Results:**

A total of 42 patients were included, 30 of them were male and 28 were immunocompetent. Most of the cases (36/42) were diagnosed in the last 5 years (2013–2017). The incidence of CL and MCL increased from 3.6/100,000 (2006–2012) to 13.58/100,000 (2013–2017). The majority of the patients (37/42) exhibited CL, in 30 cases as single lesions (30/37). Ulcerative lesions were more common in immunosuppressed patients (13/14) than in immunocompetent patients (20/28), (*P *= 0.2302). The length of lesion presence before diagnosis was 7.36 ± 6.72 months in immunocompetent patients and 8.79 ± 6.9 months in immunosuppressed patients (*P *= 0.1863). *Leishmania* DNA detection (92.3%) was the most sensitive diagnostic technique followed by Giemsa stain (65%) and histopathological examination (53.8%). Twelve patients (12/42) had close contact with dogs or were living near to kennels, and 10 of them did not present underlying conditions. Intralesional glucantime (21/42) and liposomal amphotericin B (7/42) were the most common treatments administered in monotherapy. All patients evolved successfully and no relapse was reported.

**Conclusions:**

Some interesting clinical and epidemiological differences were found in our series between immunocompetent and immunosuppressed patients. Future studies can take these results further especially by studying patients with biological therapy. Skin biopsies combining NAAT with histological techniques are the most productive techniques for CL or MCL diagnosis.

## Background

Leishmaniasis is a complex disease caused by protozoan intracellular parasites, belonging to the genus *Leishmania* (order Kinetoplastida, family Trypanosomatidae). The disease is transmitted by Phlebotominae sand flies of the genus *Phlebotomus* in the Old World (Europe, Africa and Asia) and *Lutzomyia* in America. The disease is endemic in 88 countries, but Spain is within the 48 countries in which its declaration is not mandatory. It is estimated that there is a total of 350 million people at risk of suffering from the disease with an annual incidence of approximately 0.7–1.2 million cases of cutaneous leishmaniasis (CL) [1]. In most cases, canids, especially dogs, act as a reservoir of the disease, although hares, foxes, cats, rats and other wild animals may also serve as sylvatic reservoirs [2–7]. Human data underestimate the actual prevalence of the disease due to certain limiting factors, including a discontinuous distribution in endemic areas and a large number of undiagnosed cases.

At least twenty species of *Leishmania* are responsible for the different clinical forms of the disease: CL, localized cutaneous (LCL) or diffuse cutaneous (DCL); mucocutaneous (MCL); and visceral (VL). A single skin ulcer (oriental sore) is the most common clinical form of CL with self-resolution capability depending of the immunological characteristics of the host [8]. CL caused by *L. infantum* is endemic in the Mediterranean Basin. However, anthroponotic CL caused by *L. tropica* and by *L. donovani* has been reported sporadically in different south European countries [9]. In America, *L. braziliensis* [10] produces mucous lesions on sites exposed fly bites (tongue, lips, palate, etc.) and lymphatic regional dissemination. DCL is more frequent in immunocompromised patients [11, 12].

The clinical manifestations of CL and MCL differ depending on the immunological status of the patients. In immunosuppressed patients, the presence of multiple skin lesions with torpid development are common, along with a higher recurrence rate and greater treatment difficulty compared to immunocompetent patients [13, 14]. However, most of these studies compared immunocompetent patients with HIV-infected patients with CD4 levels below 200 mm^3^ and with unusual manifestation not typical in our environment. For these reasons, we focused our study on describing and comparing clinical manifestations of CL and MCL in immunosuppressed and immunocompetent patients in a tertiary hospital of the Mediterranean basin.

## Methods

### Study design

An observational and retrospective study of patients with CL and MCL diagnosis at the La Fe University Hospital was conducted between September 2006 and December 2017. Definitive leishmaniasis diagnosis in lesions clinically compatible with CL or MCL was considered in any of the following: (i) presence of amastigotes by Giemsa stain of the lesion smear; (ii) visualization or amastigotes in skin or mucosal biopsy; or (iii) detection of *Leishmania* DNA in skin or mucosal biopsy.

Patient’s data were collected using a standardized protocol regarding demographic, epidemiological, clinical and laboratory parameters. The comorbidities studied were those implying a risk to the immune state of the patient (HIV infection, solid organ transplant, acute myeloid leukemia, illnesses in active immunosuppressant treatment, or being under biological immunosuppressive therapies). Patients diagnosed with VL were excluded.

### Samples and measurements

Needle aspirates, slit skin smear, brushings or scraping of slide edges were collected for Giemsa staining. Full depth punch biopsy from raised ledge ulcer or mucosal lesion was processed for histology and nucleic acids amplification techniques (NAAT). Histology techniques and NAAT were performed following the hospital Pathology and Microbiology Department guidelines, respectively [15].

### Statistical analysis

The incidence of CL and MCL was calculated using the population assigned to the hospital as denominator (entire population estimated of 210,000–250,000). Data are represented as the mean ± standard deviation (SD) and as the median and interquartile range (IQR). The SPSS V21.0. Statistical software (SPSS Inc, Chicago, IL, USA) was used for data analysis and the level of significance was established at *P* < 0.05. Fisher’s exact test was used to compare the categorical variables and Mann-Whitney U-test was used to compare continuous variables.

## Results

A total of 42 cases (37 CL and 5 MCL) were included during the study period; 36 patients were diagnosed in the 2013–2017 period (13.58 cases/100,000), and the remaining six between 2006–2012 (3.6 cases/100,000). The median age of the patients was 53 (24–67) years; 30 of them were male (30/42), 8 were children under 10 years (8/42), and 13 were older than 65 (13/42). Fourteen (14/42) patients were immunodepressed; autoimmune disease (*n *= 10), solid organ transplantation (*n *= 2), and cancer (*n *= 2). Most (9/10) patients with autoimmune diseases received immunosuppressant treatment. Eight of these were receiving inhibitors of the tumoral necrosis factor (anti-TNF); four were treated with infliximab, four were treated with adalimumab, and one was treated with methotrexate. One of the solid organ transplant recipients was in treatment with tacrolimus and the other with cyclosporine and mycophenolate. Focusing on MCL, all the patients were adults; three were immunosuppressed (only one was receiving adalimumab) and the other two were immunocompetent.

The most common skin manifestation in immunocompetent patients was plaque (13/28), while in immunosuppressed patients there was more likely to be a nodule (8/14). In this group, ulcerative lesions were also more common compared to the immunocompetent group (13/14 *vs* 20/28). However, no statistically significant differences were found between the two groups (*P *= 0.2302). The length of lesion appearance before diagnosis was 7.36 ± 6.72 months in immunocompetent patients and 8.79 ± 6.9 in immunosuppressed patients (*P *= 0.1863). Ulcerative lesions were reported in all cases of MCL; these were also purulent. Table 1 summarizes the clinical lesions in relation to the immunological status of patients.

**Table 1:** Characteristics of the lesion skin and the immune state of patients with CL and MCL

Variable	Group	ICP (*n *= 28)	ISP (*n *= 14)	Total (*n *= 42)
Close contact with dogs or living near to kennels		10	2	12
Age < 5 years		6	0	6
Age > 65 years		7	6	13
Male		21	9	30
Skin lesion	Papule	8	1	9
	Plaque	13	5	18
	Nodule/tumor	7	8	15
Ulceration or crust		20	13	33
Nodular lymphangitis		2	0	2
Location of CL	Head/neck	11	4	15
	Thorax	2	0	2
	Upper extremities	7	5	12
	Lower extremities	6	2	8
	Combination	0	1	1
Location of MCL	Nasal mucosa	1	1	2
	Oral mucosa	1	2	3
Number of skin lesions	Unique	25	13	38
	Multiple	3	1	4

*Abbreviations*: ICP, immunocompetent patients; ISP, immunosuppressed patients

**Fig. 1:**
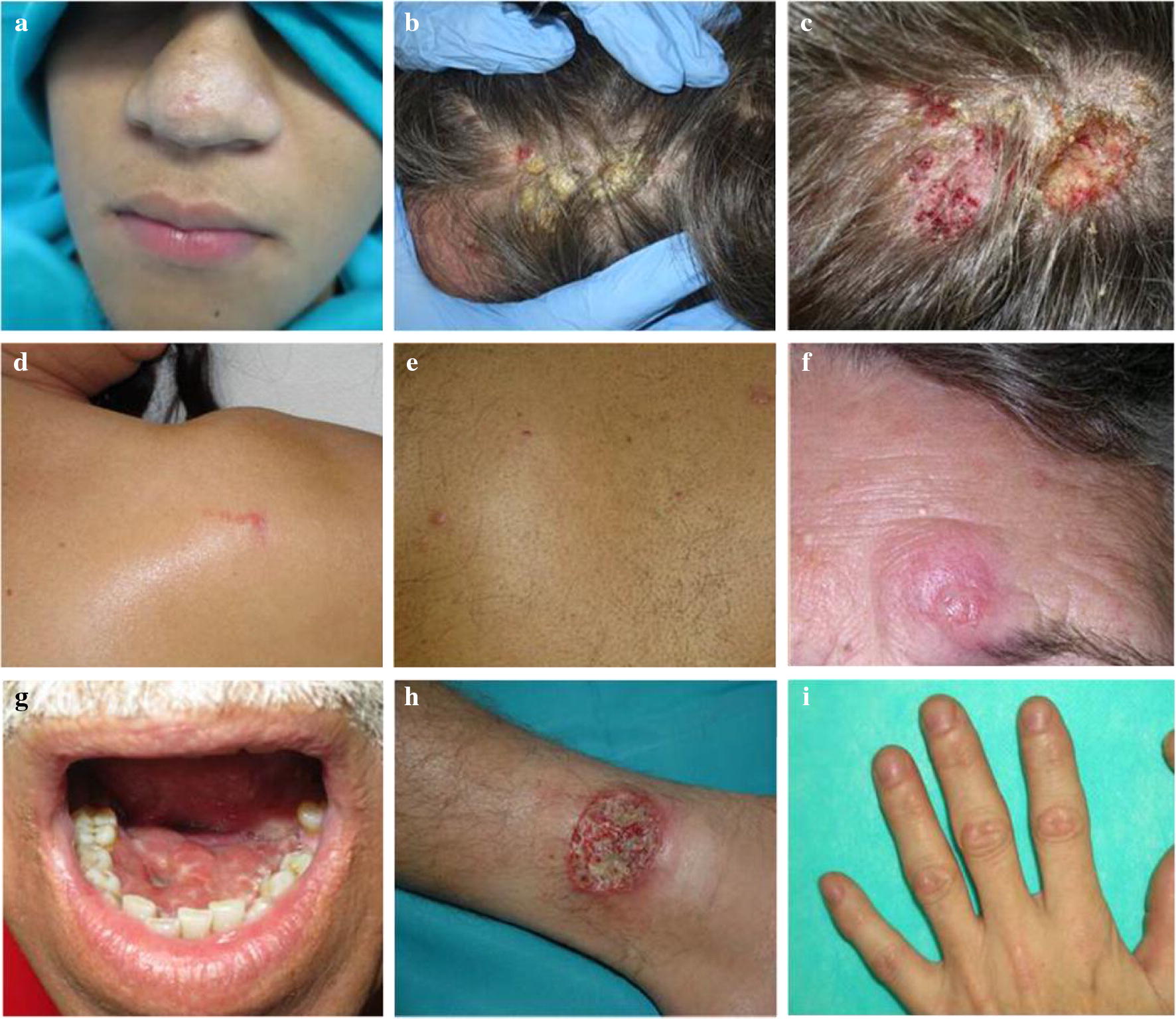
**a** Cupuliform papules with normal color in nasal top suggestive of hamartoma or anexial tumor. **b** Hiperqueratose crust plaque in scalp of patient with Crohn’s disease in treatment with infliximab suggestive of epidermoid carcinoma. **c** Previous patient after the retreat of the crusts. **d**, **e** Immunocompetent patients with orange papules on the back suggestive of sarcoidosis. **f** Left supraciliar violet nodule suggestive of skin lymphoma/pseudolymphoma. **g** Patient with rheumatoid arthritis in treatment with infliximab with a lesion compatible with epidermoid carcinoma in soil mouth. **h** Ulcerated plaque with elevated verge that might confused with a gangrenosum pyoderma. **i** Patients with papule-nodule lesions on the back of their hands suggestive of multicentric reticulohistiocytosis or dermatomyositis Gottron’s papules

CL was suspected in the initial diagnosis in twenty-seven patients (27/42), 14 as unique diagnosis (14/27), and 13 as multiple diagnosis (13/27). In 17 patients leishmaniasis was not clinically suspected. In general, no significant differences were found in the initial diagnosis between immunocompetent and immunosuppressed patients, except for sarcoidosis that was considered as an initial diagnostic only in immunocompetent patients. Table 2 shows the main differential diagnosis in CL and MCL cases. Regarding MCL cases, the initial diagnosis was epidermoid carcinoma in all the patients and leishmaniasis was not suspected in any of them. Figure 1 shows some of the lesions presented by the patients; the high heterogeneity of presentation made the initial diagnosis difficult.

**Table 2:** Main differential diagnosis in CL and MCL cases

Leishmaniasis diagnosis	Differential diagnosis	CL (*n *= 37)	MCL (*n *= 5)
Leishmaniasis (unique diagnosis)		14	0
Leishmaniasis in combination (more than one initial diagnosis in some patients)		13	0
	Mycobacteriosis	5	
	Epidermoid carcinoma	3	
	Pyoderma gangrenosum	3	
	Sarcoidosis, lymphoma, ecthyma, nodular lymphangitis	5	
Other	Epidermoid carcinoma	0	5
	Lichen, lymphoma, sarcoidosis, juvenile xanthogranuloma	10	0

Microscopical examination of lesion smear by Giemsa stain was performed in 18 patients (18/42) and was positive in 13 of these cases (13/18). Meanwhile, the histopathological study was carried out in 36 patients (36/42), and amastigotes were identified in 20 of the biopsied lesions (20/36). No significant differences (*P *= 0.3741) were found between the positive rates of both microscopy techniques. NAAT were performed in 36 patients, being positive in 33 of the cases (33/36). *Leishmania infantum* was identified in 32 cases by NAAT (32/33). In the remaining case, *L. braziliensis* was identified in a patient from South America with CL and lymphangitic involvement. In the three NAAT-negative samples, the definitive diagnosis was made by histology in two cases, and by the combination of smear and histology in the remaining patient. In 13 cases (13/42) the definitive diagnosis was only possible by NAAT, because amastigotes could not be visualized by microscopy techniques. NAAT were not carried out in six patients, in which definitive diagnosis was confirmed by Giemsa stain in five paediatric patients, and by histology in one case. The diagnostic sensitivity of NAAT was superior to histology with significant differences (*P *= 0.0013) but similar to Giemsa stain (*P *= 0.1362). Granulomatous infiltrates with abundant plasma cells were presented in 27 patients (27/42).

The most employed treatment was intralesional glucantime in monotherapy (21/42), mainly (16/21) in immunocompetent patients (*P *= 0.163). The median of treatment cycles was 2 (range 1–4), and the immunosuppressed patients needed more treatment cycles (range 3–4) (*P *= 0.2118). Liposomal amphotericin B in monotherapy was administered in 7 patients (7/42), four of them were also receiving immunosuppressive therapy; one with cyclosporine and three with anti-TNF therapy. Two of these patients stopped temporarily the anti-TNF therapy until liposomal amphotericin B treatment was finished. The remaining immunocompetent patients (3/7), were treated with this drug because one patient presented sporotrichoid pattern and the other two patients had mucosal involvement. Other treatments administered were: topical paromomycin (*n *= 2), imiquimod (*n *= 1), miltefosine (*n *= 1), combination of topical paromomycin with intralesional glucantime (*n *= 1), and combination of intralesional glucantime with liposomal amphotericin B in a patient who did not respond to four infiltrations of glucantime in monotherapy (*n *= 1). In six patients, treatment was not necessary because recovery occurred after the diagnostic biopsy. A patient returned to his home country before starting treatment, so the skin lesion resolution was unknown. Focusing in MCL cases, the majority of patients were treated with amphotericin B in monotherapy (4/5) and in the remaining patient recovery occurred after the diagnostic biopsy. All the patients were cured, and no relapse was reported. A total of 12 patients had close contact with dogs or were living near to kennels (12/42), and 10 of them did not present underlying conditions.

## Discussion

The incidence of CL and MCL in our area has showed an increase from 3.6 to 13.58 cases/100,000 inhabitants in the period of the study (2006–2017), coinciding with the data observed Spain, probably due to the higher rate of clinical suspicion and the introduction of NAAT as a diagnostic tool that is more sensitive than microscopic techniques. According to RENAVE (Red Nacional de Vigilancia de la Salud Pública), the average incidence of leishmaniasis in Spain was 0.45 cases/100,000 population in the period between 1996–2011. Valencia was one of the regions with the highest incidence during this period together with Baleares, Madrid, Andalusia and Catalonia [16]. In 2012, a large leishmaniasis outbreak, related to hares, was reported in the surroundings of Madrid city (21.54 cases/100,000 inhabitants) [17]. Furthermore, cases of CL are expected to rise in coming years in Spain, due to the increase in animal reservoirs (especially those who live free and without any veterinary supervision), immunodepressed hosts, aging population, migratory movements, and global warming [12, 18, 19].

It is estimated that approximately 7% of the canine population is infected by *L. infantum* in Spain, although in some regions, such as Valencia and Catalonia, this reaches 30% [20]. In our study, the majority of patients who had contact with dogs were immunocompetent. For this reason, the patients’ anamnesis and knowledge about the place of residence (rural areas, dumping grounds and kennels) are necessary for the early and correct diagnosis, especially in immunocompetent patients.

Although leishmaniasis is considered a disease with a higher incidence in children, most of our cases were adults, especially older than 65 years. In young children CL is more common than MCL, although VL is the main clinical presentation in this group [21, 22]. CL diagnosis is difficult in paediatric patients because children are often diagnosed with impetigo, prurigo or folliculitis [23]. Furthermore, in order to avoid the lesion biopsy in these patients, only a clinical diagnosis is usually done and the possibility of an erroneous diagnosis is high. In these cases, a lesion smear is a good option instead of a biopsy, reducing the possibility of a wrong diagnosis. In our series skin biopsy was performed only in three children (3/8).

As others, we observed a predominance of leishmaniosis in male patients [12]. In our study most patients were immunocompetent, although immunosuppression is a well-established risk factor for disease [24]. A vast number of cases of CL and MCL have been reported in HIV patients, but the experience with non-HIV immunosuppressive conditions is mostly based on case reports or small case series. The role of drugs, especially anti-TNF and new antineoplastic agents, in the risk of developing leishmaniasis is controversial. The risk seems greater in the first year of treatment and even more with the use of infliximab and adalimumab (anti-TNF monoclonal antibodies), than with no monoclonal drugs such as etanercept (dimeric fusion protein) [25, 26]. The experience in patients with other anti-interleukin biological treatments such as abatacept, rituximab (antiCD20), anti-interleukin-6 (tocilizumab), anti-interleukin-17, or its receptor (secukinumab, ixekizumab, brodalumab), or inter-leukin-12/23 (ustekinumab), is much less conclusive and future research is needed. Furthermore, the importance of steroids as predisposing factor should not be neglected, depending on the dose and the duration of the treatment [27, 28].

Ulcerative lesions were more common in immunosuppressed patients, supporting previous studies [18]. However, no statistically significant differences were found between immunosuppressed and immunocompetent patients, probably because of the small sample size. Focusing in MCL, all the patients presented ulcerative lesions which were also purulent, as the literature refers [8]. In general, delayed diagnosis is common in leishmaniasis because the lesions are usually painless [17]. In our study, the mean time from the onset of symptoms to diagnosis was greater in immunosuppressed patients, probably because the majority of immunosuppressed patients did not suffer from autoimmune dermatological diseases (psoriasis) and they were not treated by a dermatologist and the diagnosis was delayed. However, due to the small number of patient differences, there was no significant differentiation between immunocompetent and immunosuppressed patients.

The differential diagnosis of CL is quite large depending on the form of presentation. In our study, mycobacteriosis, epidermoid carcinoma and pyoderma gangrenosum were the most frequent, as reported previously [29]. Other differential diagnoses would be non-melanoma skin cancer and opportunistic or endemic fungal infections. When the skin lesion manifests as nodules, erythemato-violaceous not ulcerated, the differential diagnosis should include pseudolymphoma and cutaneous lymphoma. In these nodular forms, NAAT are crucial for definitive diagnosis, especially in those cases in which amastigotes are not visualized by microscopy.

In the paediatric population, the lesions simulated a solitary mastocytoma or a juvenile xanthogranuloma, especially if they are not ulcerated, as also described by Handler et al. [29]. Regarding MCL, in our study, epidermoid carcinoma was the unique differential diagnosis in all the patients, as reported previously [30]. However, other diseases like rhinosporidiosis or sinusitis should be also considered [30].

In La Fe University Hospital, all cases of MCL were caused by *L. infantum*, although mucocutaneous forms are mainly due to *L. braziliensis* and they are located almost exclusively in South America [31]. However, in recent years, cases of MCL in Spain and other European countries have also been reported due to *L. infantum* [32].

In our study, NAAT was the most sensitive diagnostic tool (over 90%) and significant differences were found between this technique and histology. However, no differences were found between the positive rates of both microscopy techniques and between NAAT and Giemsa, probably due to the number of patients included in our study. Microscopically, granulomatous infiltrates with abundant plasma cells are highly suggestive of leishmaniasis, and were visualized in 27 patients (27/42). Nevertheless, despite its low sensitivity, these techniques are good methods for studying inflammatory infiltrates associated with *Leishmania*. Microscopy techniques are good complements to NAAT in order to avoid the false positives due to sample contamination or cross-reactions. In our series, 16 cases were diagnosed by NAAT and the histology was compatible with infection as there were structures characterized by *Leishmania* filled macrophages and polymorphonuclear neutrophil infiltration. On the other hand, in our experience, the usefulness of Giemsa stained preparation from the lesion smear is acceptable, and should be considered as the first diagnostic step, especially in the paediatric population. Therefore, an appropriate diagnosis would be performed in the first instance based on examination of the smear of the lesion, and for Giemsa-negative cases, based on biopsy and NAAT [33].

In our patients, intralesional glucantime was the most common treatment in CL, leaving liposomal amphotericin B for MCL, for complicated CL or for immunosuppressed patients, coinciding with literature [18]. Regardless of the treatment administered and the patient’s immune status, all patients progressed satisfactorily, and no relapses were observed.

## Conclusions

The incidence of CL and MCL is increasing progressively in La Fe University Hospital as in other hospitals of the Mediterranean Region. These diseases may not be diagnosed in patients without underlying illness, although in our series 66.6% of the patients were immunocompetent. Thus, leishmaniasis epidemiology is changing. Moreover, the characteristics of the lesions and the clinical management depend on the immune status and the age of the patients. For these reasons, multidisciplinary clinical strategies, including a high clinical suspicion, and strict control of reservoirs and vectors are necessary to avoid the disease expansion in endemic countries.

## Data Availability

All relevant data are contained within the article and full datasets are available from the corresponding author upon reasonable request.

## Abbreviations

CL: cutaneous leishmaniasis
MCL: mucocutaneous leishmaniasis
NAAT: nucleic acid amplification techniques
DCL: diffuse cutaneous leishmaniasis
VL: visceral leishmaniasis
RENAVE: Red Nacional de Vigilancia de la Salud Pública
